# Chinese Wild-Growing *Vitis amurensis ICE1* and *ICE2* Encode *MYC*-Type *bHLH* Transcription Activators that Regulate Cold Tolerance in *Arabidopsis*

**DOI:** 10.1371/journal.pone.0102303

**Published:** 2014-07-14

**Authors:** Weirong Xu, Yuntong Jiao, Ruimin Li, Ningbo Zhang, Dongming Xiao, Xiaoling Ding, Zhenping Wang

**Affiliations:** 1 School of Agronomy, Ningxia University, Yinchuan, Ningxia, P.R. China; 2 Engineering Research Center of Grape and Wine, Ministry of Education, Ningxia University, Yinchuan, Ningxia, P.R. China; 3 Ningxia Engineering and Technology Research Center of Grape and Wine, Ningxia University, Yinchuan, Ningxia, P.R. China; 4 College of Horticulture, Northwest A & F University, Yangling, Shaanxi, P.R. China; Institute of Genetics and Developmental Biology, Chinese Academy of Sciences, China

## Abstract

Winter hardiness is an important trait for grapevine breeders and producers, so identification of the regulatory mechanisms involved in cold acclimation is of great potential value. The work presented here involves the identification of two grapevine *ICE* gene homologs, *VaICE1* and *VaICE2*, from an extremely cold-tolerant accession of Chinese wild-growing *Vitis amurnensis*, which are phylogenetically related to other plant *ICE1* genes. These two structurally different ICE proteins contain previously reported ICE-specific amino acid motifs, the bHLH-ZIP domain and the S-rich motif. Expression analysis revealed that *VaICE1* is constitutively expressed but affected by cold stress, unlike *VaICE2* that shows not such changed expression as a consequence of cold treatment. Both genes serve as transcription factors, potentiating the transactivation activities in yeasts and the corresponding proteins localized to the nucleus following transient expression in onion epidermal cells. Overexpression of either *VaICE1* or *VaICE2* in *Arabidopsis* increase freezing tolerance in nonacclimated plants. Moreover, we show that they result in multiple biochemical changes that were associated with cold acclimation: *VaICE1/2*-overexpressing plants had evaluated levels of proline, reduced contents of malondialdehyde (MDA) and decreased levels of electrolyte leakage. The expression of downstream cold responsive genes of *CBF1*, *COR15A*, and *COR47* were significantly induced in *Arabidopsis* transgenically overexpressing *VaICE1* or *VaICE2* upon cold stress. *VaICE2*, but not *VaICE1* overexpression induced *KIN1* expression under cold-acclimation conditions. Our results suggest that *VaICE1* and *VaICE2* act as key regulators at an early step in the transcriptional cascade controlling freezing tolerance, and modulate the expression levels of various low-temperature associated genes involved in the C-repeat binding factor (*CBF*) pathway.

## Introduction

Grapevine (*Vitis* L.) is one of the most widely cultivated fruit crops worldwide, and is of great economic importance. However, grape production is often severely limited by various biotic and abiotic stresses [1]–[5]. For example, low temperature stress greatly restricts the geographic range of grapevine cultivation, and decreases berry yield and quality. Significantly, the grapevine cultivars that currently dominate the market in terms of acreage and production of premium wines are derived from the species *Vitis vinifera*, but they tend to be very sensitive to low winter temperatures [6]. Thus, enhancing low temperature tolerance of grapevine is of great practical importance.

In this context, the current study focuses on *Vitis amurensis* Rupr., a wild grape species that is native to China and is extremely cold-tolerant [7], withstanding freezing temperature as low as −40°C [8]. *V. amurensis* therefore has great potential as an experimental system to identify mechanisms of cold tolerance and as a germplasm resource for grapevine cold-resistance breeding. Some highly cold-resistant *Vitis* cultivars have previously been produced using classical breeding methods; however, resistance to cold stress is a multigenic trait, which limits the effectiveness of using traditional breeding [9]. Therefore, understanding the mechanisms underlying tolerance and adaptation to cold stress could potentially lead to the development of new strategies for improving the yield of cold sensitive agronomic plants and expanding the geographic areas of production.

Cold acclimation has been extensively studied, resulting in considerable evidences at the molecular level that cold stress triggers a multitude of physiological responses [10]. Cold responses are complex and highly regulated via activation of signaling pathways and numerous genes encoding proteins that act directly in stress tolerance. To date, numerous cold-regulated (*COR*) genes have been functionally identified, such as antifreeze proteins, later embryo abundant (*LEA*) proteins, molecular chaperones and enzymes involved in detoxification and biosynthesis of osmoprotectants [9], [11]–[13]. Ectopic expression of some *COR* genes have been reported to result in improved cold tolerance, including studies with tobacco [14]–[16], rice [17], [18], strawberry [19] and *Arabidopsis* [20], [21]. Conversely, the expression of *Craterostigma plantagineum* or spinach *LEA* proteins in tobacco did not induce any significant changes in freezing tolerance [15], [22]. Such findings are not surprising since a clear increase in freezing tolerance is rarely obtained by expressing a single cold-induced gene, even if the end product is directly related to development of freezing tolerance.

Recent studies have shown that *Arabidopsis* cold stress tolerance can be enhanced by modulating the signaling pathways triggered by low temperature stress [23]–[25]. Such pathways include the *CBF* (C-repeat binding factor), also known as *DREB* (dehydration responsive element binding)/*ICE* (inducer of CBF expression) signaling pathway, which have also been characterized in other plant species [25]–[27]. It was reported that increased expression of the entire battery of *COR* genes resulted from overexpressing the *Arabidopsis* transcriptional activator *CBF1* [23], [24], [28], [29]. The underlying mechanism involves the CBF1 protein binding to the CRT/DRE regulatory element located in the promoters of the target genes, thereby inducing cold response metabolic pathways and enhancing cold tolerance. Jaglo-Ottosen et al. [23] further found that constitutive overexpression of *CBF1* induces expression of the *COR* genes in non-acclimated *Arabidopsis* plants and increased freezing tolerance at a whole plant level, an effect that was not observed by expressing *COR15A* alone. This further suggests that freezing tolerance is a multigenic trait involving genes with additive effects. Overexpression of *Arabidopsis CBF* genes, which reside at the nodes of regulatory networks in cold responses, were able to improve chilling/freezing tolerance in different plant species, or homologs from other plant species could enhance the freezing tolerance of transgenic *Arabidopsis* [30], [31]. Other known regulatory components include an upstream transcription factor, *ICE1* (inducer of *CBF* Expression 1), which encodes a *MYC*-type transcription factor that positively regulates *CBF3*, and which plays a critical role in cold acclimation [32]. Additionally, the *Arabidopsis* gene *AtICE2*, a positive regulator belonging to the *bHLH* family, has been shown to activate *AtCBF1* [33].

Since the discovery of the *Arabidopsis ICE* genes, homologs have subsequently been found in a variety of crop species, including wheat, rice, banana, tea, trifoliate orange and grapevines[34]–[44]. In addition, *ICE*-like proteins have been overexpressed in transgenic plants and shown to increase stress tolerance [34], [35], [37]. For example, overexpression of an *ICE*-like gene from *V. amurensis* in tobacco was reported to result in a increased cold tolerance [42], and *Arabidopsis* has been shown to become more tolerant of cold, drought and salt stresses when expressing the *V. vinifera ICE1a* or -*1b* genes [43]. In this current study, we investigated whether two members of the *ICE* gene family from a highly cold-tolerant accession of *V. amurensis* differ functionally from those previously identified from other *Vitis* species, or whether they play similar roles in activating multiple components of cold acclimation responses.

## Materials and Methods

### Plant materials, growth conditions, and cold treatment

Two-year-old Chinese wild-growing *Vitis amurensis* accession ‘Heilongjiang Seedling' potted plants developing from stem cuttings were grown in a greenhouse under natural photoperiod. For the cold treatment, plants with a uniform growth status were transferred to a chamber (LT-BIX120L, LEAD-Tech (SHANGHAI) SCIENTIFIC INSTRUMENT CO., LTD, China) at 0°C with a 16 h photoperiod (200 µmol m^−2^ s ^−1^ light) and 8 h dark. Unstressed plants were used as controls (0 h). Leaves from untreated (control) and cold-treated *V. amurensis* plants were harvested at time points 0, 1, 3, 6, 12, 24, and 48 h and immediately frozen in liquid nitrogen, prior to RNA extraction. More than 3 plants were collected and pooled for each time point, and the sampling was in triple for biological replicates.

### Structural features, phylogenetic tree and expression analysis

Total RNA derived from grapevine leaves were isolated using the plant RNA kit (Omega Bio-tek, Doraville, GA, USA), and first-strand cDNA synthesis was synthesized from 2 µg of total RNA using PrimeScript RT Reagent Kit according to the manufacturer's manual (TaKaRa, Dalian, China). Two partial lengths of *V. amurensis ICE* cDNA fragments were amplified by PCR using degenerate primers 5′-TGGACTSSTCSTCKTCGTGYTCKCC-3′ and 5′-TCATCMGCCTCRTCWWTCAAACCSGA-3′, 5′-CCTYCAGTKGGBKCACAGCCMACKCT-3′ and 5′-GCAAACCYATTGAARCAGCTGATRACMGC-3′, which were designed based on the known nucleotide sequence of *ICE* homologs from other plant species. Isolation of the full length cDNA sequences was carried out using the SMART RACE Kit (Clontech, Palo Alto, CA, USA). Amplification was performed at 94°C for 3 min; 27 cycles of 94°C for 30 s, 55 to 58°C for 30 s, and 72°C for 1 min; followed by 5 min at 72°C. The cDNA pools for 3′ and 5′ RACE were generated from total RNA extracted from cold-stressed leaves of *V. amurensis*, using the RNAprep Pure Plant Kit (Omega). Subsequently, a nested PCR was performed with the prepared cDNA pool using the adaptor primer UPM and *VaICE1* gene-specific primer (GSP) 5′-CATCCACATGTTCTGTGGCCGCAGACCAGG-3′ for 3′RACE, 5′-CTGATGACCGCTTGCTGAATGTCTAGCCCAAGGC-3′ for 5′RACE; and the *VaICE2* gene-specific primer: 5′-GCCGGCGTCAGACAGTACTGGAAGCTTAGG-3′ for 3′RACE, 5′-ATCGCTCGCACTGGAGCTTTGTCGAAGCGC-3′ for 5′RACE. Amplicons were cloned into the pMD19-T vector (TakaRa) for sequencing and the full-length cDNAs of *ICE* homologs were predicted by comparing and aligning the 5′- and 3′-RACE amplified sequences, using BioEdit software (Version 7.0.1). The putative full-length cDNAs were amplified using primers designed from the extreme 5′ and 3′ ends and 5′RACE-Ready cDNA as template, cloned to pMD-19-T (TaKaRa) to generate pMD-*VaICE1*, and pMD-*VaICE2*, and verified by sequencing. Chromosomal location prediction was performed using the BLAT server (http://www.genoscope.cns.fr/cgi-bin/vitis/webBlat) at the Genoscope Genome Browser. The molecular weights (MW) and isoelectric points (pI) of the corresponding proteins were predicted with the ProParam tool (http://www.expasy.ch/tools/pi_tool.html). Nuclear localization signals were predicted based on the predicted protein sequence using the online server (http://www.predictprotein.org/) and homolog searches were conducted with the NCBI BLAST server (http://blast.ncbi.nlm.nih.gov/Blast.cgi). Molecular model building of bHLH-ZIP domain of VaICE1 or VaICE2 was carried out using SWISS-MODEL server (http://swissmodel.expasy.org). Sequence alignment was performed using the DNAMAN software (Version 6.0, Lynnon Biosoft) and the phylogenetic tree was constructed with MEGA 5.1 software [45] using the neighbor-joining method.

To determine the expression profiles of *VaICE1* and *VaICE2* in *V. amurensis* and *Arabidopsis* plants, total RNAs were extracted as described above. After treatment with RNase-free DNase, the first-strand cDNA was synthesized using the PrimeScript First Strand cDNA Synthesis Kit (TaKaRa). Semi-quantitative RT-PCR was performed to assess the expression of *VaICE1* and *VaICE2* over a cold stress time course. The *VaICE1* cDNA was amplified using the primers 5′-ATGTTACCCAGGTCGAACGACGT-3′ and 5′- CTACAGCATACCGTGGAAGCC TG--3′, and the *VaICE2* cDNA was amplified using the primers 5′-ATGCTGTCCAGAGTGAACGGCGTC-3′ and 5′-CTACAGCATACCGTGGAAGCCTG-3′. Grapevine *GAPDH* (GenBank accession no. CB973647) was used as a loading control using the following primers: 5′-TTCTCGTTGAGGGCTATTCCA-3′ and 5′-CCACAGACTTCATCGGTGACA-3′. *Arabidopsis Actin2* (AT3G18780) was served as reference control using the following primers: 5′-CTTGCACCAAGCAGCATGAA-3′ and 5′-CCGATCCAGACACTGTACTTCCTT-3′. Three replicates were performed for each semi-quantitative RT-PCR reaction.

### Subcellular localization and transactivation activity assay

To construct green fluorescent protein (GFP) translational fusion vectors, full-length *VaICE1* and *VaICE2* cDNAs were PCR-amplified with the primers 5′-ggaattcCATATGATGTTACCCAGGTCGAACGACGT-3′ (*EcoR* I) and 5′-gcGTCGACCTACAGCATACCGTGGAAGCCTG-3′(*Sal* I), 5′-catgCCATGGATGCTGTCCAGAGTGAACGGCGTC-3′ (*Nco* I) and 5′-cgGG ATCCCTACAGCATACCGTGGAAGCCTG-3′(*BamH* I), and fused in-frame upstream of the of GFP reporter gene in the pBI221 vector. The empty vector containing only GFP sequence was used as a positive control. The isolated plasmids were concentrated to ∼1 µg/µl, and used to coat one set of gold particles for bombardment experiments. *VaICE1*::*GFP* and *VaICE2*::*GFP* fusion proteins were transiently expressed in onion (*Allium cepa*) epidermal cells using particle bombardment and subsequent localization of the proteins was performed as previously described [46]. Images were collected from the transiently transformed onion epidermal cells (n≥20) that experienced a 24-h dark culture (22°C), using a confocal laser-scanning microscope (LAM510, Carl Zeiss GmbH, Jena, Germany).

For the transcriptional activation assay, the sequences corresponding to the predicted open reading frames (ORFs) of *VaICE1,* amplified with 5′-gcTCTAGAATGTTACCCAGGTCGAACGACGT-3′ (*Xba* I) and 5′-gcTCTAGACAGCATACCGTGGAAGCCTGCCG-3′ (*Xba* I), and *VaICE2*, amplified with 5′-ccgCTCGAGATGCTGTCCAGAGTGAACGGCGTC-3′ (*Xho* I) and 5′-ggGGTACCCAGCATACCGTGGAAGCCTGCCG-3′(*Kpn* I), were fused in frame with the GAL4 DNA binding domain in the pGBKT7 vector, to make pGBKT7-*VaICE1* and pGBKT7-*VaICE2*, respectively. These constructs, as well as pCL1 (positive control) and pGBKT7 (negative control), were transformed into yeast strain AH109 cells and transformants were streaked on plates containing SD/Trp- and SD/Trp-/His-/Ade- medium. After incubation at 28°C for 3 days, the growth of the transformants were evaluated. A *β*-galactosidase assay was carried out according to the manufacturer's instructions (Clontech).

### Generation *Arabidopsis* plants overexpressing *VaICE1* or *VaICE2*

The cDNA sequences corresponding to the ORFs of *VaICE1* or *VaICE2* was amplified from 5′RACE-Ready cDNA using the following gene-specific primers containing sequences for restriction endonucleases: 5′-ccATCGATATGTTACCCAGGTCGAACGACGT-3′ (*Cla* I) and 5′-gcTCTAGACTACAGCATACCGTGGAAGCCTG-3′ (*Xba* I) for *VaICE1* and 5′-ccATCG ATATGCTGTCCAGAGTGAACGGCGTC-3′ (*Cla* I) and 5′- ccgCTCGAGCTACAGCATACCGTGGAAGCCTG-3′ (*Xho* I) for *VaICE2*. The PCR products were cloned immediately adjacent to the 3′ end of the CaMV35S promoter in the pART-CAM-S vector [47]. The resulting construct was confirmed by sequencing and transferred into *Agrobacterium tumefaciens* strain GV3101 by electroporation. *Arabidopsis* transformation was performed by the floral dip method [48]. T1 seeds were collected from individual lines and screened on 50 mg/mL kanamycin-contained medium to analyze the segregation of the resistant phenotype. T2 kanamycin-resistant seeds were harvested from non-segregating families and confirm the kanamycin-resistant phenotypes. T3 homozygous lines were validated by RT-PCR using the primers as above described in grapevine expression analysis, and further used for all experiments.

### Stress tolerance assay

For the cold treatment, surface-sterilized T3 homozygous seeds of vector-carrying control and *VaICE1*/*2*-overexpressing *Arabidopsis* lines were germinated and grown on MS medium supplemented with 50 mg/L kanamycin for one week, then transferred to pots containing a mixture of perlite: sand: peat (1∶1∶1, v/v) for two weeks. The three-week-old seedlings of the transgenic plants overexpressing *VaICE1* and *VaICE2* (n = 50) and control plants (n = 50) were transferred to the chamber (LT-BIX120L) at 0°C under the condition as above-described in grapevine for 0, 6, 12, 24, or 48 h. The freezing tolerance assay was performed by transferring 3-week-old seedlings (n = 50) grown in pots to the pre-chilled chamber (LT-BIX120L) at −6°C for 8 h under continuous dim light conditions (2.5 µmol m^−2^ s^−1^) and subsequently returning them to normal conditions. Survival rates of plants were evaluated after 7 days. The freezing treatment experiment was performed in triplicate.

### Determination of electrolyte leakage, and malondialdehyde and proline content

Three-week-old seedlings from the empty vector control *Arabidopsis* plants (n = 20), *VaICE1* and *VaICE2* T3 transgenic lines (n = 20) were subjected to the cold stress treatment described above and leaves were harvested to determine three biochemical features associated with cold tolerance: electrolyte leakage was assessed by ion leakage analysis as previously described [49]; proline content was determined using acid-ninhydrin reagent and acetic acid [50]; and malondialdehyde (MDA) content was measured using the thio-barbituric acid (TBA) method as previously described [51].

### Analysis of cold-responsive genes regulated by *VaICE1* or *VaICE2*

Total RNA was extracted from vector-carrying control and T3 transgenic *Arabidopsis* leaves treated at 0°C for 0, 3, 12, 24 and 48 h using the plant RNA kit (Omega) following the manufacturer's instructions. cDNA was synthesized from 2 µg of total RNA using the PrimeScript RT Reagent Kit with the oligo (dT)18 primer, according to the manufacturer's instructions (TakaRa) and quantitative real-time PCR was performed using the SYBR Premix Ex ™ TaqII kit (TakaRa) on an iCycler iQ5 thermal cycler (Bio-Rad). The reactions were carried out in triplicate in 96-well plates (25 µl/well) in a mixture containing 12.5 µl 2×SYBR Premix Ex ™ TaqII, 1 µl each of primer (10 µM in stock), 1 µl template cDNA and 9.5 µl ddH2O. Two-step real-time PCR reactions were performed under the following conditions: 95°C for 10 s, followed by 40 cycles of denaturation at 94°C for 15 s, annealing and extension at 57°C for 30 s, and data acquisition at 57°C for 15 s. Three replicate PCR amplifications were performed for each sample. Transcript levels of AtICE1, AtICE2, AtCBF1, AtCBF2, AtCBF3, AtCOR15A, AtCOR47, AtRD29A and AtKIN1 were measured in VaICE1 or VaICE2 overexpressing or empty vector control plants. The amount of transcript for each gene, normalized to the internal reference Atactin2, was analyzed using the 2-△△Ct method [52]. Oligos used for real-time PCR were: 5′- CTTCCATCCGTTGACACCTAC -3′ and 5′-CTCTAGCTTGCTGGCCTTTAG-3′ for AtICE1; 5′-TCCACAAACGCTGTCTTACC-3′ and 5′-GTTCACTGCCTTTCCTTCTCT-3′ for AtICE2; 5′-GAGACGATGGTGGAAGCTATTT-3′ and 5′-AGCATGCCTTCAGCCATATTA-3′ for *AtCBF1*; 5′-GACCTTGGTGGAGGCTATTT-3′ and 5′-ATCCCTTCGGCCATGTTATC-3′ for *AtCBF2*; 5′-GACGTTGGTGGAGGCTATTT-3′ and 5′-AGCATCCCTTCTGCCATATTAG-3′ for *AtCBF3*; 5′-GGCGTATGTGGAGGAGAAAG-3′ and 5′-CCCTACTTTGTGGCATCCTTAG-3′ for *AtCOR15A*; 5′-GGCTGAGGAGTACAAGAACAA-3′ and 5′-ACAATCCACGATCCGTAACC-3′ for *AtCOR47*; 5′-GCAATGTTCTGCTGGACAAG-3′ and 5′- TCCTTCACGAAGTTAACACCTC-3′ for *AtKIN1*; 5′-GCTTTCTGGAACAGAGGATGTA-3′ and 5′-CGACTCTTCCTCCAACGTTATC-3′ for *AtRD29A*. Data analysis shown was obtained from three biological replicates.

## Results

### Characterization of two *ICE* homologs from *V. amurensis*

**Figure 1:**
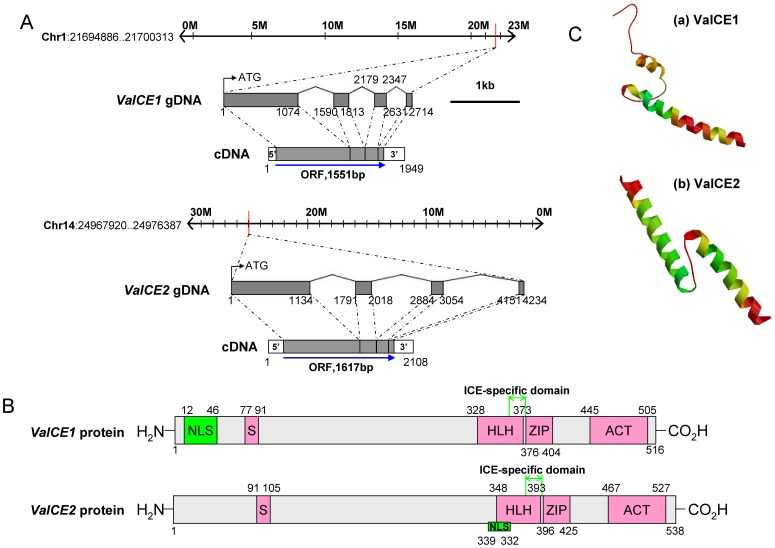
Location and structure of the *VaICE1,2* genes. (A) Gene structure and locus. Chromosomal localization of *VaICE1,2* genes were predicted in *V. vinifera* cv. Pinot Noir clone P40024 genome. *VaICE1* comprises four exons spanning 2.7 kb with a predicted 1,551 bp ORF, mapping to Chromosome 1 and position 21694886–21700313. *VaICE2* contains four exons spanning 4.2 kb and has a predicted 1,617 bp ORF, which is mapped to Chromosome 14 and position 24967920–24976387. Nucleotide numbers are indicated above the gene structure. (B) Schematic protein structures of VaICE1 and VaICE2 with the N-terminal domain containing a S-rich motif and the C-terminal domain represented by helix-loop-helix (HLH), ICE-specific domain, Zipper region (ZIP), and ACT_UUR_ACR-like (ACT) domain. The putative nuclear localization signals (NLS) are indicated as green boxes. Codon numbers are indicated above the protein structures. (C) The predicted tertiary structures of the bHLH-ZIP domains of the (a) VaICE1 and (b) VaICE2 proteins evaluated by the SWISS-MODEL sever.

Two *ICE*-orthologs, designated *VaICE1* (GenBank accession no. KC815984) and *VaICE2* (GenBank accession no. KC815985), were isolated from leaves of the cold-tolerant Chinese wild-growing *V. amurensis* accession ‘Heilongjiang Seedling’. The complete cDNA of *VaICE1* is 1,949 bp, comprising a 97 bp 5′UTR, 301 bp 3′UTR and a 1,551 bp ORF that was predicted to encode a 516 amino acid protein with a MW of 55.5 kDa and a pI of 5.30. The complete cDNA of *VaICE2* is 2,108 bp, comprising a 214 bp 5′UTR, 277 bp 3′UTR and a 1,617 bp ORF that was predicted to encode a 538 amino acid protein with a MW of 58.38 kDa and a pI of 5.05. A sequence alignment showed that the two proteins differ significantly from each other (60% amino acid similarity). Chromosome location of the two *ICE* homologs in the *V. vinifera* cv. Pinot Noir clone P40024 genome suggested that *VaICE1* is mapped on chromosome 1, spanning 2714 bp, while *VaICE2* is mapped on chromosome 14 and spans 4234 bp (Fig. 1A). Analysis of structural properties revealed that the predicted VaICE1 or VaICE2 protein possesses the typical features of ICE proteins, including a serine-rich region (S-rich), a basic helix-loop-helix (bHLH) domain, an ICE-specific domain [38], a zipper region (ZIP) and an ACT_UUT-ACR-like domain (Fig. 1B). An interPro scan suggested that both proteins belong to the *MYC*-like *bHLH* family of transcription factors, and in support of this, they contain nuclear location signals (NLSs) at position 12–46 aa for VaICE1 and 339–362 aa for VaICE2 (Fig. 1B). The predicted three dimensional structures of the bHLH-ZIP domain of VaICE1 and VaICE2 were distinct, and were more similar to the structures of DIMER (PDB ID: 1r05) and HETERO DIMER (PDB ID: 2ql2), respectively (Fig. 1C).

**Figure 2:**
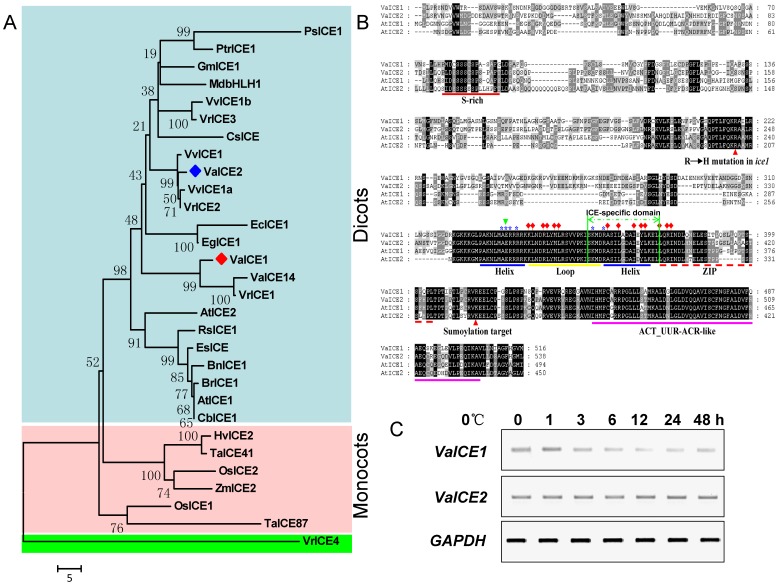
Phylogenetic tree, alignment and expression profiles of *VaICE1*,*2*. (A) Phylogenetic tree based on the deduced amino acid sequences of ICE from a range of plant species. A neighbor-joining tree, with the following predicted ICE protein sequences, with GenBank accession numbers listed in parentheses: *V. amurensis* (VaICE1, AGP04217; VaICE2, AGP04218; VaICE14, ADY17816), *V. vinifera* (VvICE1, AFI49627; VvICE1a, AGQ03810; VvICE1b, AGQ03811), *V. riparia* (VrICE1, AGG34704; VrICE2, AIA58705; VrICE3, AIA58706; VrICE4, AIA58707), *Arabidopsis thaliana* (AtICE1, NP_189309; AtICE2, NP_172746), *Brassica napus* (BnICE1, AEL33687), *Brassica rapa* subsp. Chinensis (BrICE1, ACB70963), *Capsella bursa-pastoris* (CbICE1, AAS79350), *Camellia sinensis* (CsICE, ACT90640), *Eucalyptus camaldulensis* (EcICE1,ADY68776), *Eucalyptus globules* (EgICE1, AEF33833), *Eutrema salsugineum* (EsICE, ACT68317), *Glycine max* (GmICE1, ACJ39211), *Hordeum vulgare* (HvICE2, ABA25896), *Malus x domestica* (MdbHLH1, ABS50251), *Oryza sativa* (OsICE1, Os11g0523700; OsICE2, Os01g0928000), *Populus suaveolens* (PsICE1, ABF48720), *Populus trichocarpa* (PtrICE1, ABN58427), *Raphanus sativus* (RsICE1, ADY68771), *Triticum aestivum* (TaICE41, ACB69501; TaICE87, ACB69502), *Zea mays* (ZmICE2, ACG46593), was produced by ClustalX 2.0 alignment followed by tree construction using MEGA 5.0 with 100 bootstrap tests. The branch support values are indicated. The length of the scale bar corresponds to 5 substitutions per site. (B) Comparison of ICE amino acid sequences from *V. amurensis* (VaICE1,2) and *Arabidopsis thaliana* (AtICE1,2). Deduced amino acid sequences were aligned using ClustalW. Sequences and accession numbers are shown for the following: *Vitis amurensis* (VaICE1, KC815984; VaICE2, KC815985) and *A. thaliana* (AtICE1, AAP14668; AtICE2, AAO63441). Residues in black and gray regions indicate identical and similar residues, respectively, between isoforms. Four predicted domains are labeled: a S-rich motif, a basic-helix-loop-helix-leucine zipper (bHLH-ZIP) region, *ICE*-specific domain [38] and a ACT-UUR-ACR-like domain. The red triangles indicate the position of the mutation isolated by Chinnusamy et al. [32] and Kanaoka et al. [54], and the residue targeted for sumoylation by SIZ1[53]. Green triangle indicates E-box/N-box specificity site [69], blue asterisks indicate core residues for DNA binding sites [70], and red diamonds indicate dimerization interface/polypeptide binding sites [70]. (C) Expression profiles of *VaICE1* and *VaICE2* during a cold stress time-course experiment. Semi-quantitative RT-PCR was used to determine *VaICE1* and *VaICE2* transcript levels in cold treated grapevine leaves at indicted times. Grapevine *GAPDH* was used as a loading control.

Phylogenetic analysis of various ICE amino acid sequences indicated the existence of two major groups (Fig. 2A). VrICE4 is phylogenetically distinct from the other groups in which they could be clearly classified into dicot and monocot specific subgroups. Our phylogetic data demonstrated VaICE1 and VaICE2 fell into different clades of the dicot subgroup, with VaICE1 being closely related to the previously reported VaICE14 (98.7% identity) from *V. amurensis* [42], VrICE1 (97.7% identity) from *V. ripara* [44], whereas VaICE2 being closely similar to VrICE2 (99.4% identity) from *V. ripara* [44], VvICE1 (98.9% identity) and VvICE1a (98.7% identity) from *V. vinifera* [43]. In addition, VvICE1b shared 98.72% identity with VrICE3, which belongs to another clade of dicot subgroup. Comparison of VaICE1,2 and their homologs AtICE1 and AtICE2 from *Arabidopsis*, as determined by 49∼60% sequence similarity, revealed they share highly conserved regions in the bHLH DNA binding domain and ACT domain in their C-terminal regions (Fig. 2B). In contrast, only a moderate sequence conservation was found in the ZIP domains between *Vitis* and *Arabidopsis* ICE proteins. A potential sumoylation site previously identified in *Arabidopsis* ICE proteins [53] was also observed in VaICE2, and the S-rich region, which has been suggested to be a site of phosphorylation [32], [54] was present in either VaICE1 or VaICE2. Finally, the R236 residue that substituted with H236 in the *Arabidopsis ice1* mutant [32], causing a loss of ICE function, was shown to be present in the two *V. amurensis* ICE proteins (Fig. 2B).

To gain insight into the biological functions of VaICE1 and VaICE2, semi-quantitative RT-PCR was used to examine their expression profiles in leaves of *V. amurensis* over a cold stress time-course (Fig. 2C). *VaICE2* transcript levels were constant over the time course, while a rapid induction of *VaICE1* expression was observed after 1 h, followed by a gradual decline from 3 h to the minimum level after 12 h, and finally a return to background levels at the end of the time course. These results suggest that *VaICE1* and *VaICE2* may be involved in cold stress responses.

### *VaICE1*/*VaICE2* is nuclear-localized and can act as transcriptional activators in transient assays

**Figure 3:**
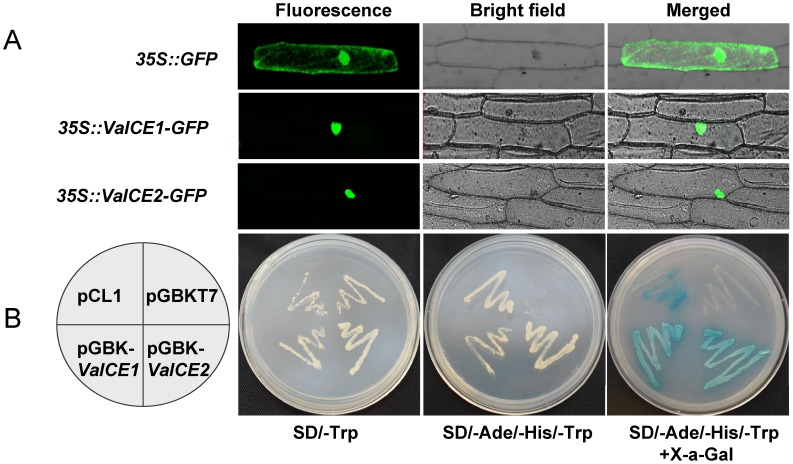
Nuclear localization and transcriptional activation assay of *VaICE1,2*. (A) Confocal imaging of the VaICE1 and VaICE2 transiently expressed in onion epidermal cells as GFP fusion proteins, driven by the 35S promoter. GFP alone was used as a control. Left hand panels show dark field images of green fluorescence, middle panels show the morphology of the cells in bright field and the right hand panels show the merged images. (B) Fusion proteins of pGBKT7-*VaICE1*, pGBKT7-*VaICE2*, pCL1 (positive control) and pGBKT7 (negative control) were expressed in the yeast strain AH109. Transformants were incubated on SD/Trp- and SD/Trp-/His-/Ade- to assess their growth and tested for *β*-galactosidase activity.

Sequence analysis showed that VaICE1 and VaICE2 have putative NLSs, suggesting that they target the mature proteins to the nucleus. To test this, VaICE1 and VaICE2 were transiently expressed as translational fusions at the N terminus of GFP in onion (*Allium cepa*) epidermal cells. Confocal imaging showed that GFP alone (control) was present in the cytoplasm and nucleus, as expected, whereas cells transformed with either *VaICE1::GFP* or *VaICE2::GFP* showed strong fluorescence exclusively in the nucleus (Fig. 3A).

Yeast one hybrid assays were carried out to determine whether VaICE1 and VaICE2 possess transactivation activity. As shown in Fig. 3B, yeast transformants with pCL1 (positive control), pGBKT7-*VaICE1* and pGBKT7-*VaICE2* grew well on the SD/Trp- medium, but also grew normally on the SD/Trp-/His-/Ade-medium, and exhibited fairly strong *β*-galactosidase activity. In contrast, transformants carrying the negative control (pGBKT7) did not grow on the SD/Trp-/His-/Ade-medium and could not show *β*-galactosidase activity. Thus, VaICE1 and VaICE2 have transactivation activities in yeasts.

### Over-expression of *VaICE1* or *VaICE2* in *Arabidopsis* increases cold tolerance

**Figure 4:**
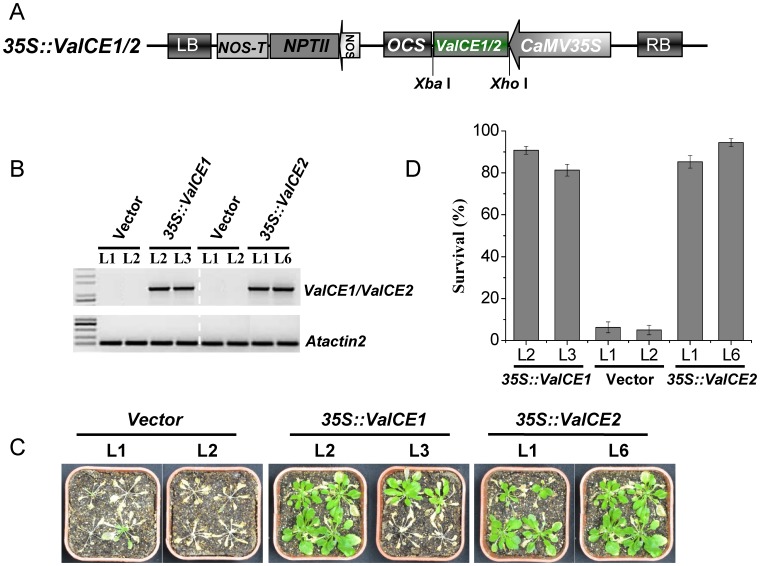
Freezing tolerance evaluation of *35S::VaICE1*,*2* transgenic *Arabidopsis* plants. (A) A schematic map of the T-DNA region of *35S::VaICE1*,*2* fusion constructs employed for *Arabidopsis* transformation. RB, right border; LB, left borders; CaMV35S, Cauliflower mosaic virus 35S promoter; OCS, octopine synthetase terminator; NOS, nopaline synthase promoter; NPTII, Kanamycin resistance gene; NOS-T, nopaline synthase terminator. (B) RT-PCR was used to assess the transcript abundance of *VaICE1* or *VaICE2* in the transgenic *Arabidopsis* plants. *Atactin2* was used as a reference control (C) 3-week-old plants were treated at −6°C for 8 h and then transferred back to normal conditions for recovery. Photographs were taken after 7 d of recovery. (D) Survival rates of plants exposed to −6°C. Average survival rates and standard errors were calculated using the results of three separate experiments with 50 seedlings per line for each freezing stress.

To determine whether *VaICE1* or *VaICE2* enhances cold stress tolerance, *Arabidopsis* WT-type Col-0 was transformed with constructs carrying empty vector, *VaICE1*- or *VaICE2*-coding regions under the control of constitutive 35S promoter (Fig. 4A). Expression of *VaICE1* or *VaICE2* transgens in these T2 lines was confirmed by semi-quantitative RT-PCR, and two *VaICE1* (L2 and L3) and two *VaICE2* transgenic T3 homozygous lines (L1 and L6) with similar transcript levels (Fig. 4B) were selected for further analyses. These two independent transgenic lines over-expressing *VaICE1* and *VaICE2*, together with the corresponding empty vector-transformed control lines, were subjected to a whole plant freezing assay to evaluate plant survival after freezing treatment (Fig. 4C). The freezing test consisted of exposing 3- week-old transgenic and control plants to a temperature of −6°C for 8 h, before recovery at normal temperatures. The *VaICE1* and *VaICE2* transformed plants exhibited less freezing damage and increased survival rates compared with empty vector control plants after a 7 d recovery period (Fig. 4D). Only ∼5% of the control plants (L1 and L2) survived the treatment, while the *VaICE1*-overexpressing plants exhibited survival rates of 91% for L2 and 81% for L3, and 85% for L1 and 95% for L6 in *VaICE2*-overxpressing plants (Fig. 4D). No obvious differences in phenotype were observed between the empty vector control plants and *VaICE1* or *VaICE*2 transgenic plants grown under normal conditions. These results suggest that *VaICE1* or *VaICE2* overexpression leads to enhanced freezing tolerance.

### Over-expression of *VaICE1* or *VaICE2* affects electrolyte leakage as well as proline and MDA metabolism

**Figure 5:**
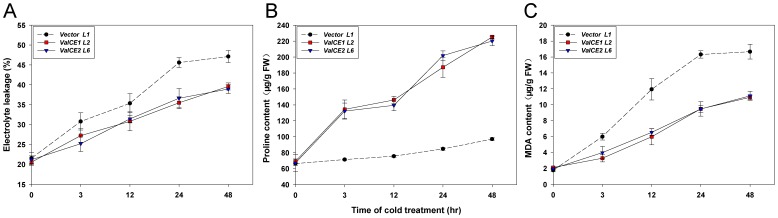
Effect of *VaICE1*, *2* expression in *Arabidopsis* on levels of (A) electrolyte leakage, (B) proline and (C) malondialdehyde (MDA). Three-week-old *Arabidopsis* plants of vector-carrying control, *VaICE1* L2, and *VaICE2* L6 were grown at 0°C for the time indicated. Leaves were collected to assess electrolyte leakage and free proline and MDA. Each value is the mean ± SD of three replicates in case of electrolyte leakage, proline and MDA content (6 seedlings each) for the indicated time points.

To investigate the physiological and biochemical factors that might contribute to the improved cold tolerance of the transgenic plants, MDA and proline content were evaluated over a time course of exposure to cold stress (0°C), as well as electrolyte leakage, which is commonly used as an index of membrane injury [23], [55]. A representative experiment comparing the empty vector control L1 and *35S::VaICE1* L2 and *35S::VaICE2* L6 transgenic plants is shown in Fig. 5. Under non-acclimation conditions, electrolyte leakage of all tested lines did not vary greatly i.e. between 22% in the control (L1) and 21% in the *VaICE1*-(L2) and *VaICE2*-(L6) overexpressing lines (Fig. 5A). Electrolyte leakage increased with the prolonged cold stress and reached 47% in the control and 40% and 39% in *VaICE1* and *VaICE2* lines, respectively, after 48 h.

Proline accumulation in plants has been associated with a wide variety of environmental stresses, and confers stress tolerance by facilitating osmotic adjustment, protecting proteins and membranes and quenching reactive oxygen species [56], [57]. Significantly, for this study, increases in proline levels have been well-documented in *Arabidopsis* and other plants during cold acclimation [58], [59]. Similar changes were observed in both *VaICE1* and *VaICE2* overexpressing *Arabidopsis* lines (Fig. 5B), since similar levels of proline were detected in the transgenic and wild type plants without cold stress, but substantial proline accumulation occurred in cold-treated *VaICE1* and *VaICE2* transgenic lines. After 48 h of cold stress, the proline contents in the leaves of *VaICE1* or *VaICE2* transgenic lines were ∼2.3-fold higher than those of the control plants (Fig. 5B).

MDA is also considered an indicator of plant oxidative stress and structural integrity of the membranes in response to low temperature [60], [61]. Under non-acclimation conditions, all tested lines had similar MDA levels ranging from 1.76–2.11 µg/g FW (Fig. 5C). However, MDA accumulation was considerably less in the *VaICE1* or *VaICE2* transgenic lines than in the controls during the cold stress time-course. These results indicated that *VaICE1* and *VaICE2* overexpression resulted in increased levels of proline, but decreased levels of MDA and reduced levels of electrolyte leakage.

### *VaICE1* or *VaICE2* positively regulate cold-induced gene expression

**Figure 6:**
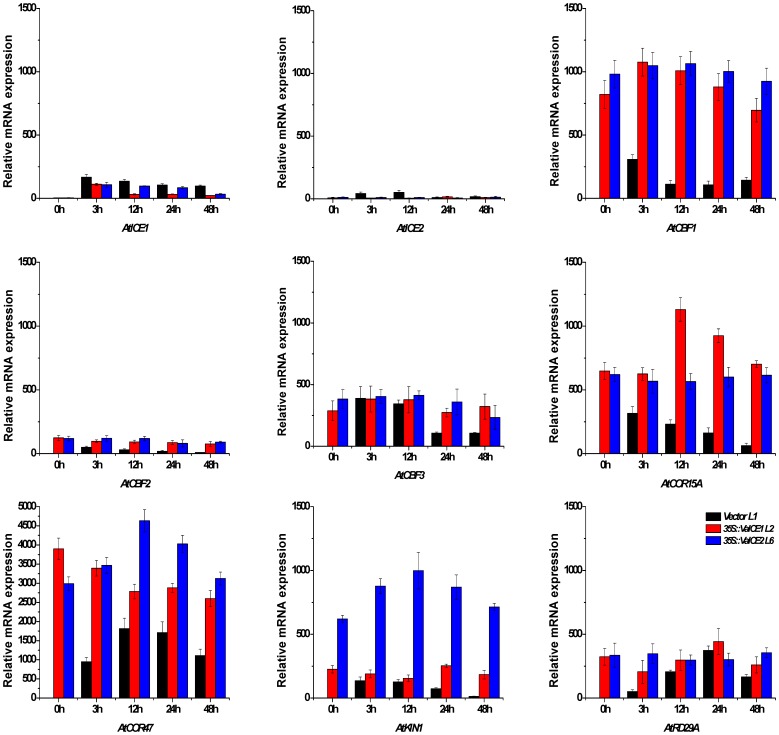
Effect of *VaICE1,2* overexpression in *Arabidopsis* on transcript levels of genes involved in the cold stress pathway. qRT-PCR analysis using leaves from control *Arabidopsis* L1, *35S::VaICE1* L2 and *35S::VaICE2* L6 plants. Three-week-old plants were grown at 0°C for the time indicated. The tested genes were *AtICE1*, *AtICE2*, *AtCBF1*, *AtCBF2*, *AtCBF3*, *AtCOR15A*, *AtCOR47*, *ATKIN1* and *AtRD29A*. *Atactin2* was used as a reference control. Each value is the mean of three replicates.

To further identify molecular components associated with the *VaICE1* and *VaICE2* mediated stress tolerance that we observed in the *35S::VaICE1* L2 and *35S::VaICE2* L6 transgenic *Arabidopsis* lines, the expression patterns of genes in the *ICE*-*CBF* pathway were evaluated by real-time PCR (Fig. 6). Under control conditions, *AtICE1* or *AtICE2* expression was relatively low in either the empty vector control or *VaICE1*- and *VaICE2*- overexpressing plants, respectively. However, overexpression of *VaICE1* or *VaICE2* resulted in a reduction in transcript levels compared with the control during the cold stress time course. The possible effect of *VaICE1* or *VaICE2* on the expression of downstream genes through transcriptional regulation was also investigated (Fig. 6). Under control conditions, transcripts of none of the three *CBF* genes was detected in the control, but higher or similar level *CBF* transcript levels were present in the *VaICE1* or *VaICE2* overexpression lines. Expression of the *CBF* genes showed a general decline in the control plants starting at 12 h of cold stress. Starting at 3 h, *CBF1* and *CBF2* transcript levels were considerably greater in the transgenic lines than in the control plants, while a substantially greater expression in the transgenic lines was not observed for *CBF3* until 24 h. Expression of the *CBF* downstream target genes (*COR15A*, *COR47*, *KIN1* and *RD29A*) were also investigated. *COR47* had extremely high expression in both transgenic lines and the vector control compared with the other genes, and *COR15A* exhibited higher expression in the transgenic lines compared with the control after 48 h. The *KIN1* gene was expressed at considerably higher levels in the *VaICE2* overexpression than that in the *VaICE1* overexpression line and the vector control, while no substantial difference in *RD29A* expression was seen among the three different genotypes. These results suggest that *VaICE1* and *VaICE2* positively regulate the expression of the *CBF* genes by differentially controlling down-stream genes in response to cold stress, which in turn likely contributes to freezing tolerance.

## Discussion

In the current study, two putative *ICE*-orthologs, *VaICE1* and *VaICE2*, from the highly cold-tolerant species *V. amurensis*, were structurally analyzed and functionally tested. We report that these two genes encode *MYC*-type *bHLH* transcription factors that are nuclear-localized, and are candidate regulators of the *CBF* gene expression during cold stress. Reports on *ICE*-orthologs in three genotypes of *Vitis* species [42]–[44] might indicate that there are multiple *ICE*-like genes in grapevine. However, sequence comparisons revealed that none of the mRNA sequences that were previously reported [42], [43] completely matched the two sequences identified here (Fig. S1 in File S1). Thus, *Vitis ICEs* were categorized on the basis of sequence similarity (Fig. S2A in File S1), phylogenetic clustering (Fig. S2B in File S1) and genetic locus (Table S1 in File S1). Our results provide evidence of the presence of 4 polymorphic loci for the identified ICE-like genes in grapevine. *VaICE1*, *VaICE14* and *VrICE1* are alleles from which they are located in grapevine chromosome 1, whereas *VaICE2*, *VrICE2*, *VvICE1* and *VvICE1a* are alleles, mapping to chromosome 14. Additionally, the other independent cluster that *VvICE1b* and *VrICE3* involved are alleles, which are located in chromosome 17. Of these three distinct categories, very few, and mostly conserved, substitutions were observed for each category, but it appears that *VrICE4* is phylogenetically distinct from the above-mentioned three categories and located in chromosome 18. A detailed sequence assay on *VrICE4* revealed that it lacked the typical S-rich motif of ICE proteins, presented 3 amino acid mutations (A-T, A-T, and E-D) in ICE-specific domain, and exhibited remarkable sequence difference in ACT_UUR_ACR-like (ACT) domain (Fig. S2A in File S1). Moreover, result from FLAGdb^++^ database prediction indicated that *VrICE4* has the best match with GSVIVG01009234001 with functional annotation as DNA binding protein rather than inducer of *CBF* expression 2 (Table S1 in File S1) when compared with other grapevine ICE proteins. Therefore, further investigation on *VrICE4* is still required to confirm its role as *ICE*-like transcription factor.

Evidence from structural and phylogenetic analyses further suggested that *VaICE1* and *VaICE2* may have different properties when involved in cold stress. This is also suggested by their expression profiles (Fig. 2C), which showing that they are both constitutively expressed, but that the expression of *VaICE1* is affected by cold treatments. With the availability of the reported expression data on grapevine *ICE* genes [42]–[44], a comparison of cold responses of these genes on different genotypes revealed that *VaIC1*, *2*, *VvICE1a*, *b* and *VrICE1-4* were all detected before and after cold treatment, which is most consistently observed to be constitutively expressed in other plants [32], [34], [40]. However, *VaICE14* is a notable exception where the transcript was not detected under non-stress condition but under low temperature [43]. One possible explanation for such difference has been proposed [44], in which this discrepancy might be attributed to the different genotypes and sampling materials derived from different cultural ways. An additional effort in validation of the expression profile of this gene before and after cold stress in repeated trials is required to clarify its expression property.

Many of the physiological and biochemical changes that occur during cold acclimation are directly correlated to an up-regulation of *COR* gene expression, which is activated by the constitutive expression of the *ICE* transcription factor. Liu et al. [62] found that overexpression of *AtICE1* in cucumber was sufficient to increase chilling tolerance and to simultaneously alter the levels of several cold responses associated factors (e.g., free proline, MDA and soluble sugars). Li et al. [43] discovered that the survival rates of *VvICE1a* and *VvICE1b*-overexpressing *Arabidopsis* lines were significantly higher than those of the wild type under cold stress. Similarly, overexpression of *Solanum lycopersicum ICE1* was reported to improve tolerance of chilling stress in tomato plants, as indicated by differences in electrolyte leakage [37]. In our study, overexpression of either *VaICE1* or *VaICE2* in transgenic *Arabidopsis* plants increased freezing tolerance at the whole-plant level, based on survival rates (Fig. 4B, C), which correlated with increased accumulation of proline, and a reduction in MDA and electrolyte leakage (Fig. 5A-C). These results suggest that although an endogenous *ICE*-*CBF* pathway is present in *Arabidopsis* system and that enables the ectopic *VaICE1,2* transgene expression to influence the plants' freezing-tolerance capacity. There are also considerable evidences to suggest that proline [63]–[65], MDA [14], [66], [67], and electrolyte leakage [25], [68], contribute to an enhancement of freezing tolerance. Our results confirmed that the greater tolerance of *VaICE1* or *VaICE2*-overexpressing plants to cold stress positively linked to elevated proline levels, and negatively correlated to the reduction of MDA content as well as electrolyte leakage. However, these data did not reveal a difference between the *VaICE1* and *VaICE2* transgenic lines. It is important to note that plants overexpressing *VaICE1* or *2* had similar levels of proline and MDA contents relative to the vector-carry controls under normal growth conditions (0 h), but upregulated in proline level and downregulated in MDA content under time-course cold stresses. We therefore assume that the commonly observed accumulation of free proline and MDA under non-acclimation are partially determined by proline biosynthesis gene (e.g. *P5CS*, d-1-pyrroline-5-carboxylate synthetase) or MDA reductase gene itself property with a relatively low expression, but also induced by the expression of a transcription factor under cold-stressed conditions. Additionally, ionic leakage from the cells is considered as an indicator that the semipermeable nature of the plasma membrane has been lost, at least transiently, in response to freezing. This is probably the main cause for the similar electrolyte leakage observed in controls and *VaICE1*-/*VaICE2*-overexpressing lines under non-stressed conditions.

We propose that our analyses of the *VaICE1* or *VaICE2* overexpressing *Arabidopsis* plants do not contradict the general cold acclimation model [32], [43], but rather suggest a more integrated circuitry involved in cold acclimation signaling. Chinnusamy et al. [32] reported that over-expression of *AtICE1* increased the expression of *AtCBF2*, *AtCBF3* and cold-regulated genes (*CORs*) under cold stress. In contrast, Fursova et al. [33] found that over-expression of *AtICE2* only resulted in dominant changes in *AtCBF1* transcription levels after cold acclimation, while Badawi et al. [34] showed that over-expression of wheat *ICE* genes in *Arabidopsis* induced a higher expression of *AtCBF2*, *AtCBF3* and some *COR* genes only after cold acclimation. Our data suggest that the expression of *AtICE1* or *AtICE2* was slightly lower in the *VaICE1* and *VaICE2* overexpressing *Arabidopsis* plants. One possible explanation for this is that co-suppression of the endogenous gene occurred in the target plant, but we suggest that the *V. amurensis* and *Arabidiopsis ICE1* or *ICE2* cDNA nucleotide sequences are not highly homologous (64.6–69.9%). The observation that several downstream genes were significantly induced in *VaICE1*- or *VaICE2*-overexpressing lines might suggest that the constitutive expression of the transgenes affects the expression of the endogenous gene. Thus, as *VaICE1* and *VaICE2* mRNA levels are abundant in the transgenic plants, competition at a post-transcriptional level could explain this slight decrease in endogenous *AtICE1* or *AtICE2* transcript levels. We speculate that another signal transduction pathway may exist in the *CBF*/*DREB1* gene regulatory network that is stimulated by *VaICE1* or *VaICE2*. It should be noted that the activation of the expression of these downstream genes differed between the *VaICE1* and *VaICE2* overexpressing lines (Fig. 6). The enhanced tolerance of *35S::VaICE1* and *35S::VaICE2* plants coincides with an up-regulation of the stress-responsive genes *CBF1*, *COR15A* and *COR47*. However, some differences were seen in the expression patterns between the transgenic lines, and particular in the case of *KIN1*, which suggests functional differences between *VaICE1* and *VaICE2*. In the case of *VvICE1a* and *VvICE1b* transgenic *Arabidopsis* lines, both contribute to the modulation of *AtRD29A* and *AtCOR47* in response to cold stress [43]. Together with the above-mentioned examples, these differences in targeting to the downstream genes are likely due to a varying number of *ICE* homologs in different plant species [32], [33], [40]–[44]. However, it has not yet been established which of the identified grapevine *ICE* genes specifically control the different sets of cold-responsive genes. A detailed comparison of the expression of these *ICE* genes and their target genes through microarray analysis might help address this issue.

Taken together, *VaICE1* and *VaICE2* are two previously unreported *ICE*-like transcription factors from *V. amurensis*, whose regulatory roles in cold acclimation are suggested by the results presented here. Both genes may act as positive regulators to increase the levels of cold-responsive genes in transgenic lines under cold stress. Moreover, *VaICE1* and *VaICE2* influence cold stress-related factors such as electrolyte leakage, and levels of proline and MDA, thereby alleviating damage by ROS and enhancing osmotic protection. Our data may help elucidate the cold-acclimation pathways of *Vitis* species and more ultimately guide the design of strategies for improving the stress tolerance of agricultural crops.

## Supporting Information

File S1 **Contains the files: Figure S1** **Multiple alignment of the mRNA sequences of** ***ICE*** **-homologous from different** ***Vitis*** **species.** Identical nucleotide sequences are highlighted on a black background while white boxes indicate at least three identical nucleotides. The GenBank accession numbers are reported as follows: *VaICE1* (KC815984), *VaICE2* (KC815985), *VaICE14* (HM231151), *VvICE1* (JQ707298), *VvICE1a* (KC831748), *VvICE1b* (KC831749), *VrICE1* (KF994961), *VrICE2* (KF994962), *VrICE3* (KF994963) and *VrICE4* (KF994964). **Figure S2** **Protein sequence similarity and phylogenetic clustering of ICE from three different** ***Vitis*** **species.** (A) Protein sequence alignment of 10 grapevine ICEs. Identical residues are outlined in black. Amino acids are numbered on the right. (B) Phylogenetic tree based on the deduced amino acid sequences of ICEs from three different *Vitis* genotypes. A maximum-likelihood phylogenetic tree of the amino acid sequences of ICE from *V. amurensis* (VaICE1, AGP04217; VaICE2, AGP04218; VaICE14, ADY17816), *V. vinifera* (VvICE1, AFI49627; VvICE1a, AGQ03810; VvICE1b, AGQ03811), and *V. riparia* (VrICE1, AGG34704; VrICE2, AIA58705; VrICE3, AIA58706; VrICE4, AIA58707) is constructed by MEGA 5.0 with 1000 bootstrap tests. The branch support values are indicated. The length of the scale bar corresponds to 0.05 substitutions per site. **Table S1 Feature lists of BLAST results or queries of ten grapevine** ***ICE*** **genes available in FLAGdb^++^.** A Blastp search with an E-Value of 1.E-50 was performed on *V. vinifera* using the ten grapevine ICE proteins from *V. amurensis* (VaICE1, AGP04217; VaICE2, AGP04218; VaICE14, ADY17816), *V. vinifera* (VvICE1, AFI49627; VvICE1a, AGQ03810; VvICE1b, AGQ03811), and *V. riparia* (VrICE1, AGG34704; VrICE2, AIA58705; VrICE3, AIA58706; VrICE4, AIA58707) as query.
(DOC)
