# Supplementary material for: Chinese Wild-Growing Vitis amurensis ICE1 and ICE2 Encode MYC-Type bHLH Transcription Activators that Regulate Cold Tolerance in Arabidopsis
Source: PLoS One. 2014 Jul 14;9(7):e102303. doi: 10.1371/journal.pone.0102303 (PMC4096504; doi:10.1371/journal.pone.0102303)
Supplement: File S1 — Contains the files: Figure S1 Multiple alignment of the mRNA sequences of ICE -homologous from different Vitis species. Identical nucleotide sequences are highlighted on a black background while white boxes indicate at least three identical nucleotides. The GenBank accession numbers are reported as follows: VaICE1 (KC815984), VaICE2 (KC815985), VaICE14 (HM231151), VvICE1 (JQ707298), VvICE1a (KC831748), VvICE1b (KC831749), VrICE1 (KF994961), VrICE2 (KF994962), VrICE3 (KF994963) and VrICE4 (KF994964). Figure S2 Protein sequence similarity and phylogenetic clustering of ICE from three different Vitis species. (A) Protein sequence alignment of 10 grapevine ICEs. Identical residues are outlined in black. Amino acids are numbered on the right. (B) Phylogenetic tree based on the deduced amino acid sequences of ICEs from three different Vitis genotypes. A maximum-likelihood phylogenetic tree of the amino acid sequences of ICE from V. amurensis (VaICE1, AGP04217; VaICE2, AGP04218; VaICE14, ADY17816), V. vinifera (VvICE1, AFI49627; VvICE1a, AGQ03810; VvICE1b, AGQ03811), and V. riparia (VrICE1, AGG34704; VrICE2, AIA58705; VrICE3, AIA58706; VrICE4, AIA58707) is constructed by MEGA 5.0 with 1000 bootstrap tests. The branch support values are indicated. The length of the scale bar corresponds to 0.05 substitutions per site. Table S1 Feature lists of BLAST results or queries of ten grapevine ICE genes available in FLAGdb++. A Blastp search with an E-Value of 1.E-50 was performed on V. vinifera using the ten grapevine ICE proteins from V. amurensis (VaICE1, AGP04217; VaICE2, AGP04218; VaICE14, ADY17816), V. vinifera (VvICE1, AFI49627; VvICE1a, AGQ03810; VvICE1b, AGQ03811), and V. riparia (VrICE1, AGG34704; VrICE2, AIA58705; VrICE3, AIA58706; VrICE4, AIA58707) as query. (DOC) [file pone.0102303.s001.doc]

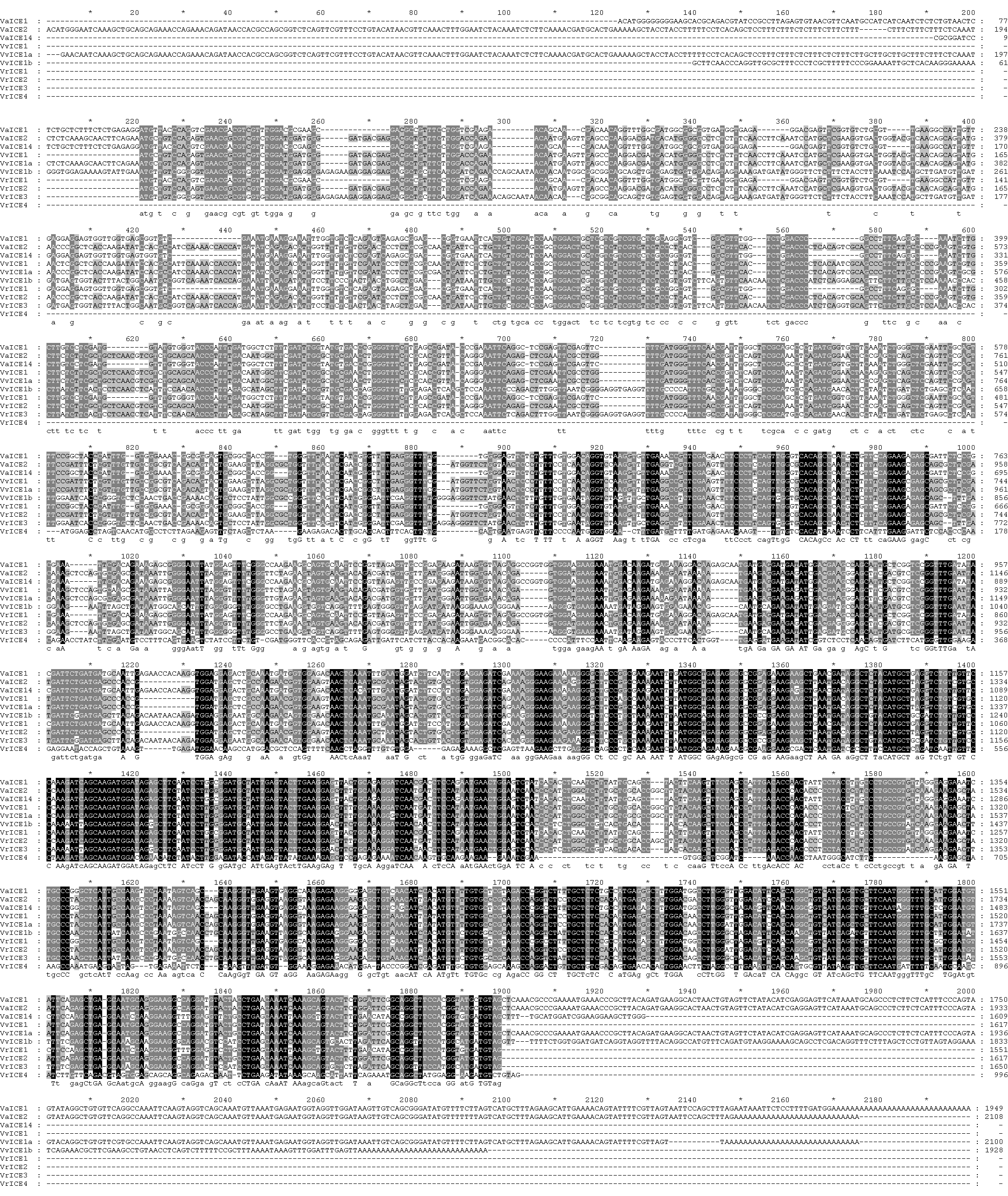


**Figure S1** **Multiple alignment of the mRNA sequences of *ICE*-homologous from different *Vitis* species.** Identical nucleotide sequences are highlighted on a black background while white boxes indicate at least three identical nucleotides. The GenBank accession numbers are reported as follows: *VaICE1* (KC815984), *VaICE2* (KC815985), *VaICE14* (HM231151), *VvICE1* (JQ707298), *VvICE1a* (KC831748), *VvICE1b* (KC831749), *VrICE1* (KF994961), *VrICE2* (KF994962), *VrICE3* (KF994963) and *VrICE4* (KF994964).


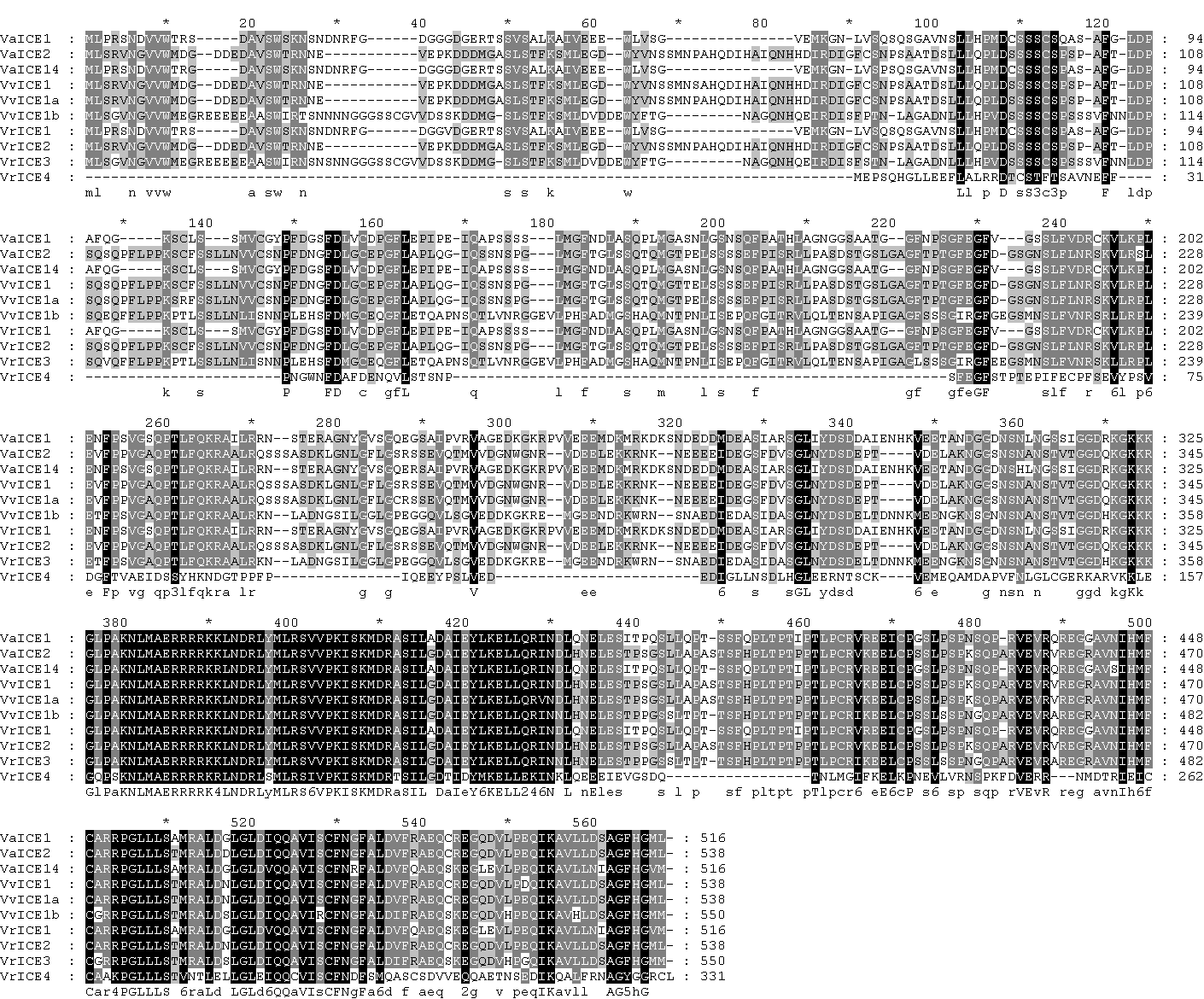


**A**

**B**

**Figure S2** **Protein sequence similarity and phylogenetic clustering of ICE from three different *Vitis* species.** (A) Protein sequence alignment of 10 grapevine ICEs. Identical residues are outlined in black. Amino acids are numbered on the right. (B) Phylogenetic tree based on the deduced amino acid sequences of ICEs from three different *Vitis* genotypes. A maximum-likelihood phylogenetic tree of the amino acid sequences of ICE from *V. amurensis* (VaICE1, AGP04217; VaICE2, AGP04218; VaICE14, ADY17816), *V. vinifera* (VvICE1, AFI49627; VvICE1a, AGQ03810; VvICE1b, AGQ03811), and *V. riparia* (VrICE1, AGG34704; VrICE2, AIA58705; VrICE3, AIA58706; VrICE4, AIA58707) is constructed by MEGA 5.0 with 1000 bootstrap tests. The branch support values are indicated. The length of the scale bar corresponds to 0.05 substitutions per site.

**Table S1 Feature lists of BLAST results or queries of ten grapevine *ICE* genes available in FLAGdb^++^.** A Blastp search with an E-Value of 1.E-50 was performed on *V. vinifera* using the ten grapevine ICE proteins from *V. amurensis* (VaICE1, AGP04217; VaICE2, AGP04218; VaICE14, ADY17816), *V. vinifera* (VvICE1, AFI49627; VvICE1a, AGQ03810; VvICE1b, AGQ03811), and *V. riparia* (VrICE1, AGG34704; VrICE2, AIA58705; VrICE3, AIA58706; VrICE4,

AIA58707) as query.

| **Query** | **Gene Name** | **EST/**  **cDNA** | **TM domains** | **PFAM** | **Function** | **SNP_probe** | **Chromosome** |
| --- | --- | --- | --- | --- | --- | --- | --- |
| VaICE1(e=2.54295e-147) | GSVIVG01004940001 | 13 | 0 | 1 | inducer of CBF expression2 | 0 | VVI01 |
| VaICE2(e=2.58193e-158) | GSVIVG01032998001 | 12 | 1 | 2 | inducer of CBF expression2 | 1 | VVI14 |
| VaICE14(e=1.34526e-156) | GSVIVG01004940001 | 13 | 0 | 1 | inducer of CBF expression2 | 0 | VVI01 |
| VvICE1(e=6.79455e-159) | GSVIVG01032998001 | 12 | 0 | 1 | inducer of CBF expression2 | 1 | VVI14 |
| VvICE1a(e=4.70538e-160) | GSVIVG01032998001 | 12 | 0 | 1 | inducer of CBF expression2 | 1 | VVI14 |
| VvICE1b(e=0) | GSVIVG01008637001 | 1 | 0 | 1 | inducer of CBF expression2 | 1 | VVI17 |
| VrICE1 (e=7.3646e-163) | GSVIVG01004940001 | 13 | 0 | 1 | inducer of CBF expression2 | 0 | VVI01 |
| VrICE2(e=0)  VrICE3(e=0)  VrICE4(e=2.50547e-107) | GSVIVG01032998001  GSVIVG01008637001  GSVIVG01009234001 | 12  1  2 | 0  0  0 | 1  1  1 | inducer of CBF expression2  inducer of CBF expression2  DNA binding protein | 1  1  1 | VVI14  VVI17  VVI18 |
